# Supplementary material for: Transcriptomic Insights into Mechanisms of Early Seed Maturation in the Garden Pea (Pisum sativum L.)
Source: Cells. 2020 Mar 23;9(3):779. doi: 10.3390/cells9030779 (PMC7140803; doi:10.3390/cells9030779)

# Correlation Network on module data ( dmap )

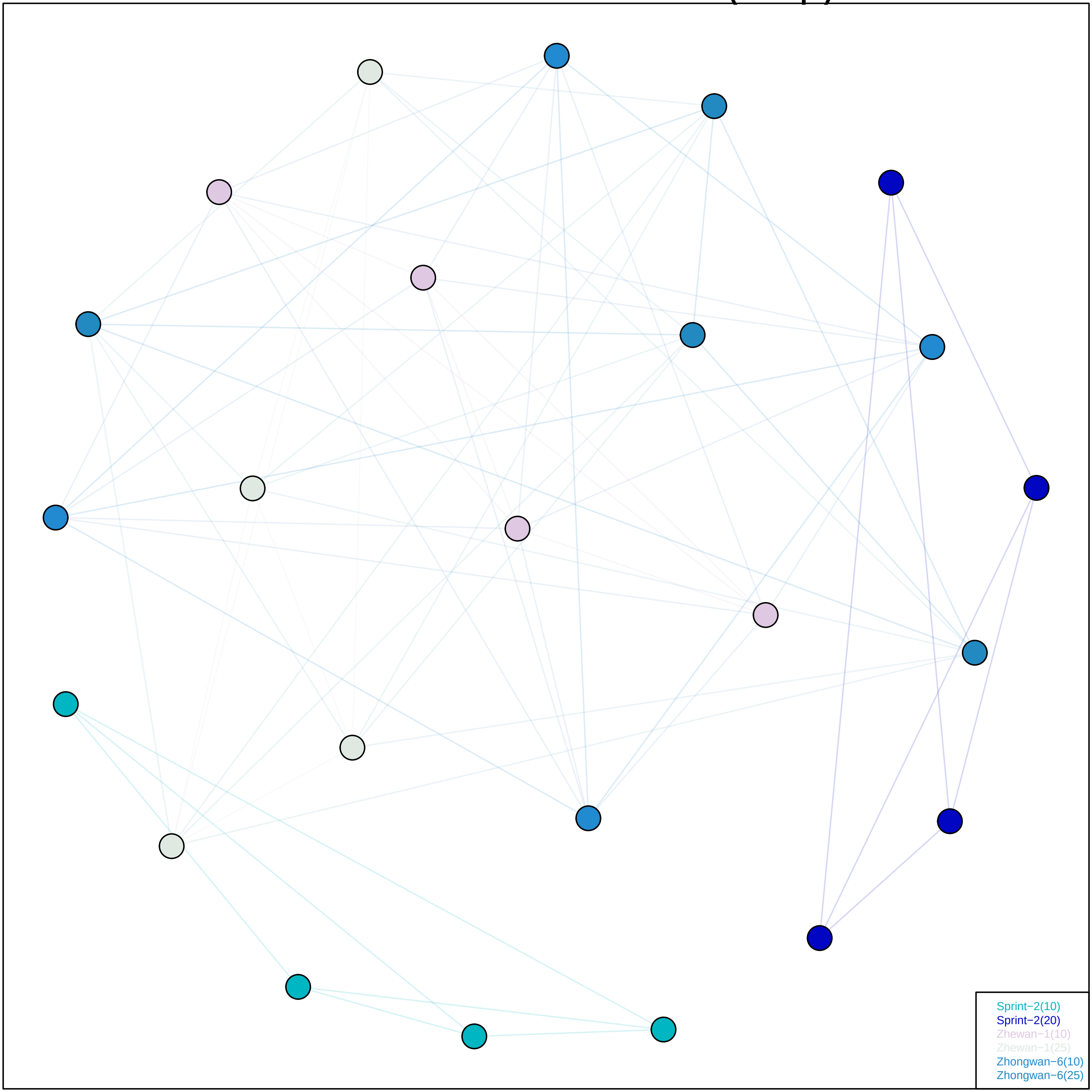

# Correlation Network & silhouette scores on module data ( dmap )

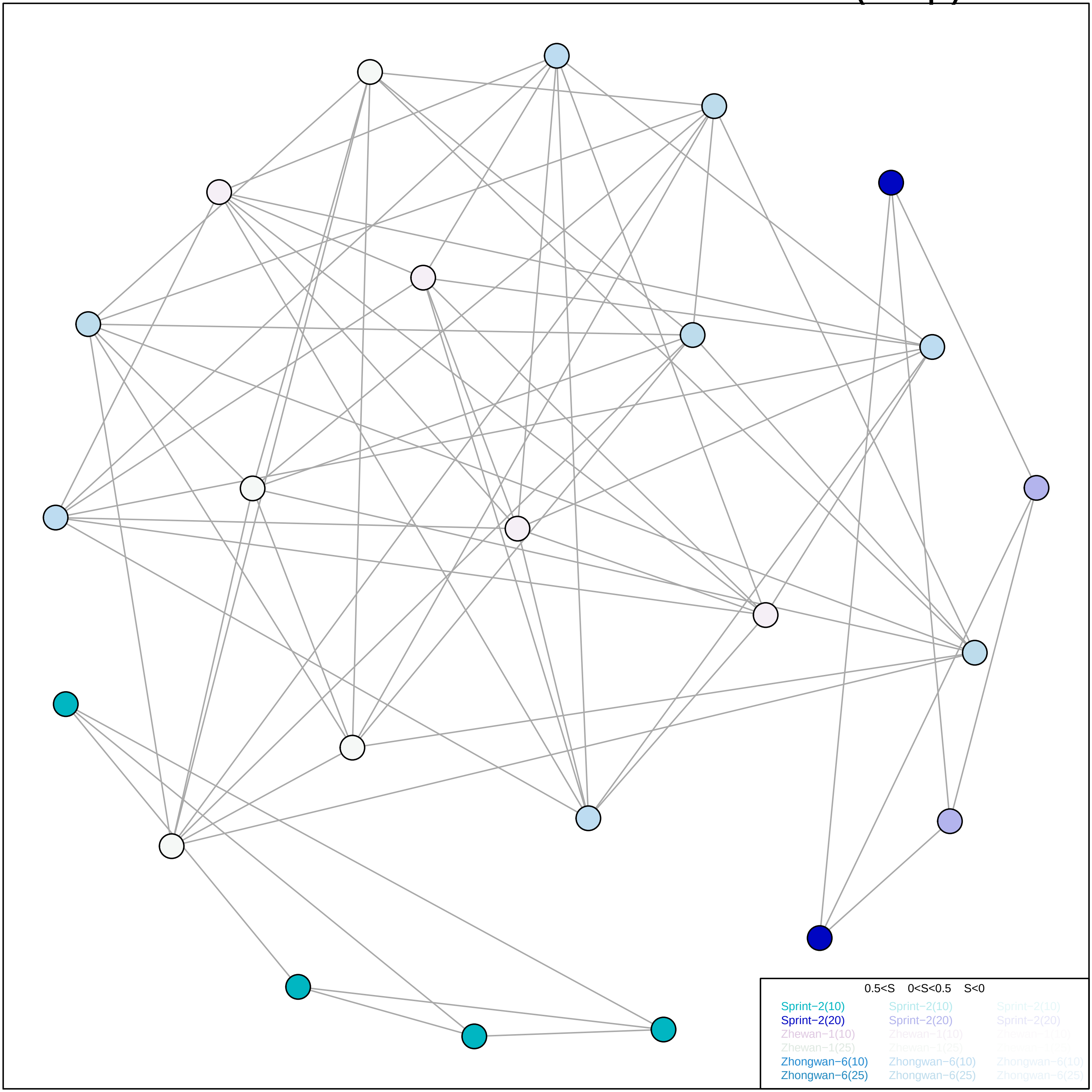

**Correlation Network on module data ( dmap )**

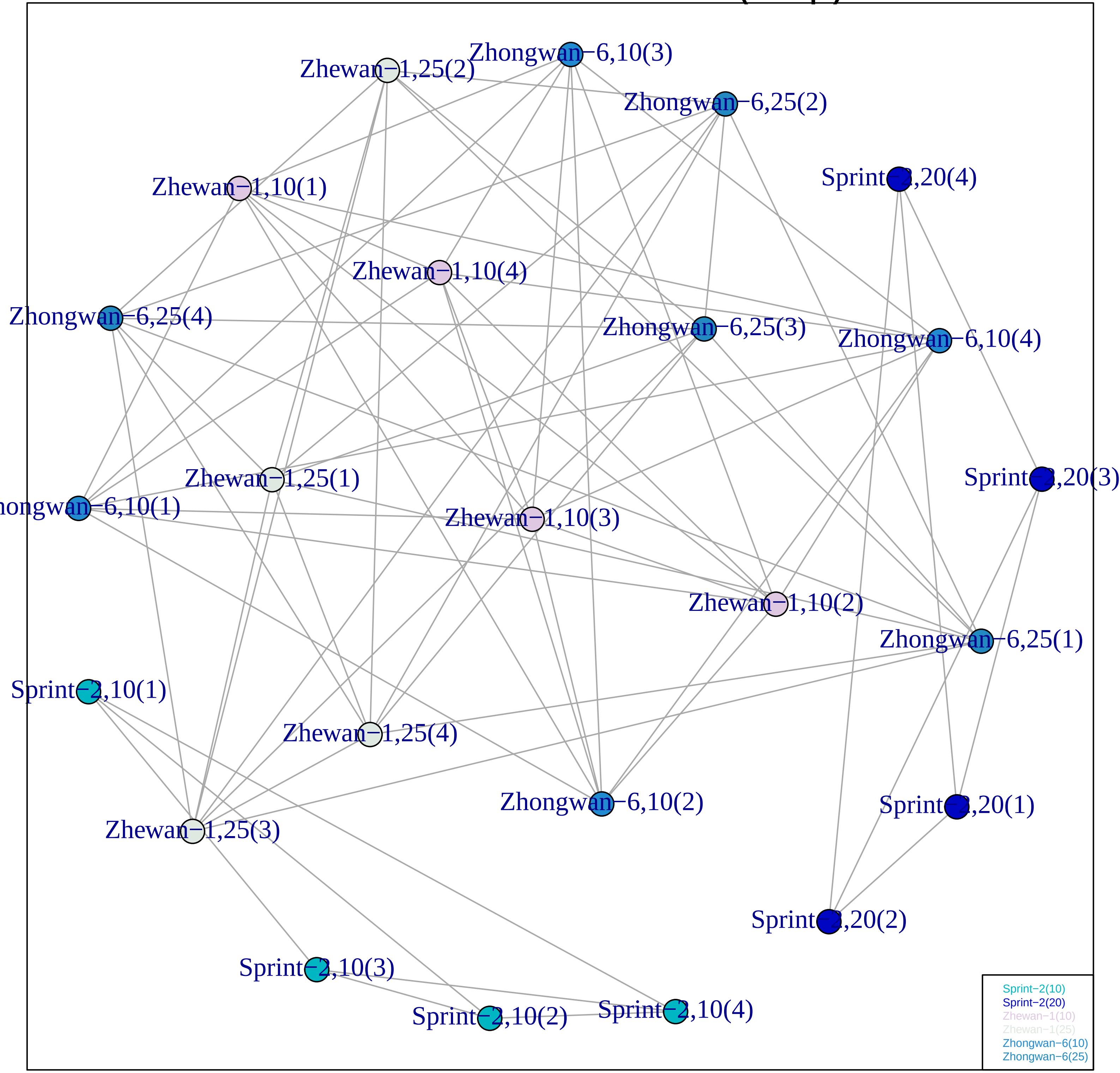

# Correlation Network on metagene data

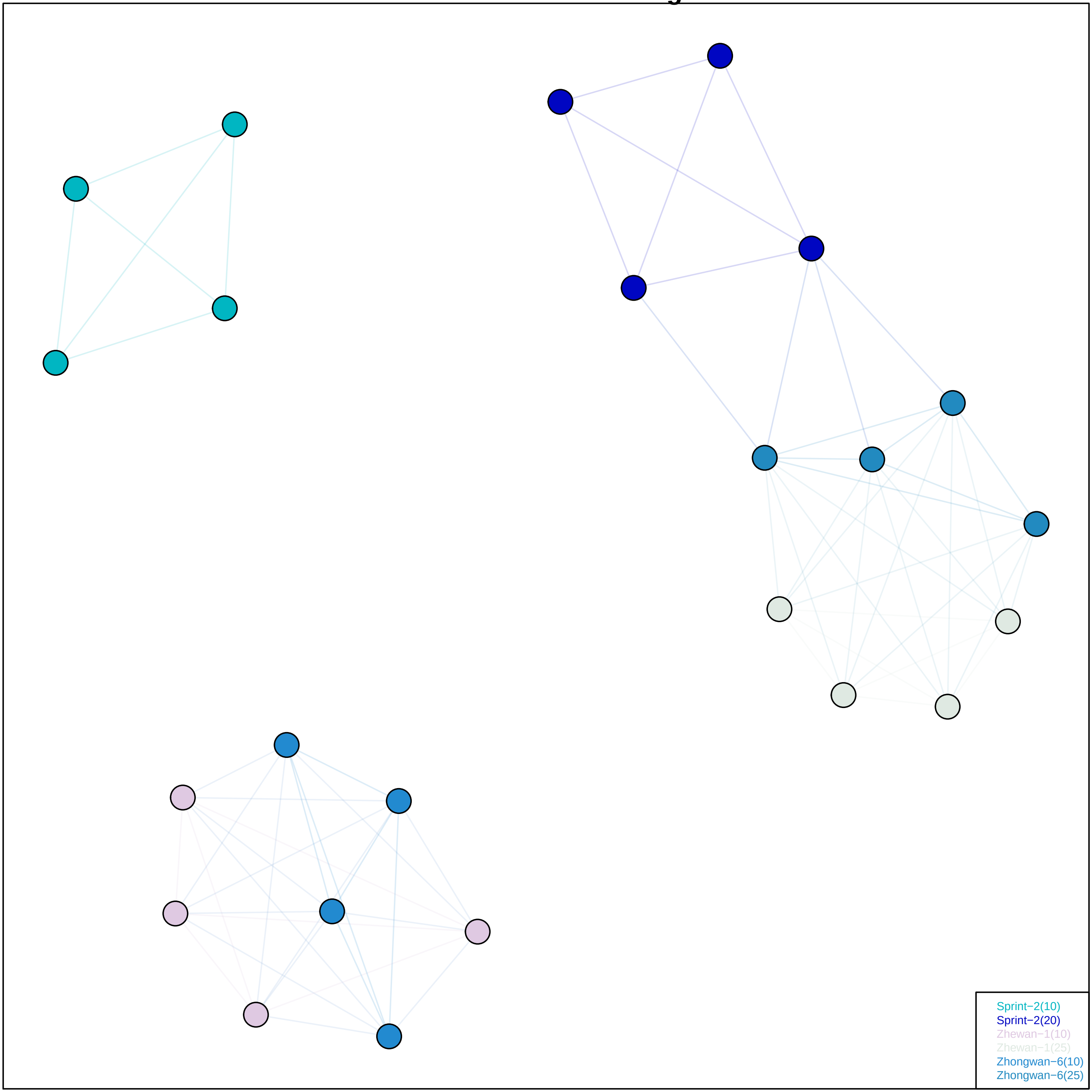

## Correlation Network & silhouette scores on metagene data

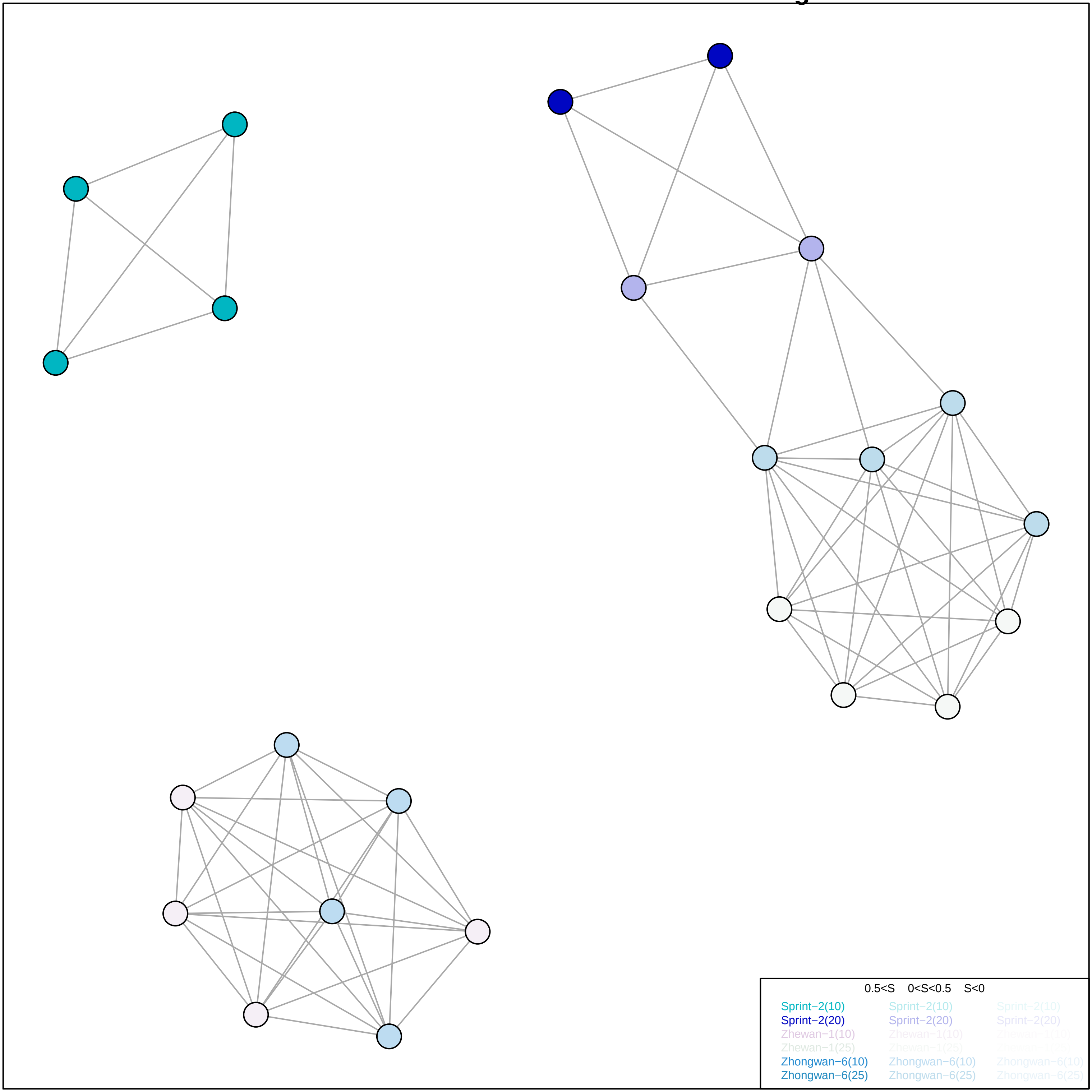

**Correlation Network on metagene data**

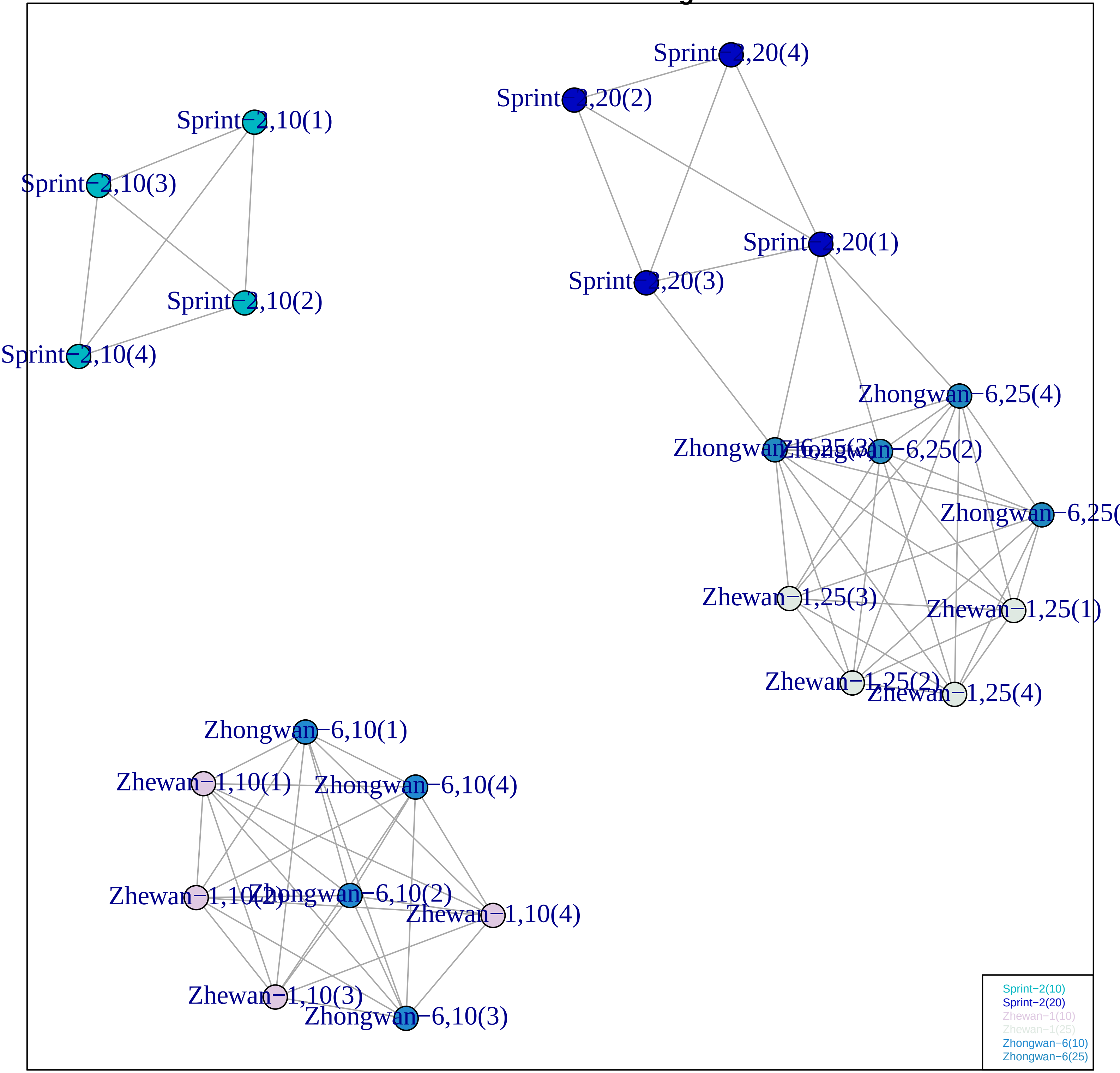

Supplement: Supplementary file 1 [file cells-09-00779-s001.zip › Supplementary materials/FileS4.zip!/FileS3/TFs - Results/Sample Similarity Analysis/Correlation Network.pdf]
